# Molecular Ecology of Hypersaline Microbial Mats: Current Insights and New Directions

**DOI:** 10.3390/microorganisms4010006

**Published:** 2016-01-05

**Authors:** Hon Lun Wong, Aria Ahmed-Cox, Brendan Paul Burns

**Affiliations:** 1 School of Biotechnology and Biomolecular Sciences, The University of New South Wales, Sydney 2052, Australia; h.l.wong@unsw.edu.au (H.L.W.); a.ahmed-cox@unsw.edu.au (A.A.-C.); 2 Australian Centre for Astrobiology, University of New South Wales, Sydney 2052, Australia

**Keywords:** hypersaline mats, microbial diversity, niche differentiation, microbial dark matter

## Abstract

Microbial mats are unique geobiological ecosystems that form as a result of complex communities of microorganisms interacting with each other and their physical environment. Both the microorganisms present and the network of metabolic interactions govern ecosystem function therein. These systems are often found in a range of extreme environments, and those found in elevated salinity have been particularly well studied. The purpose of this review is to briefly describe the molecular ecology of select model hypersaline mat systems (Guerrero Negro, Shark Bay, S’Avall, and Kiritimati Atoll), and any potentially modulating effects caused by salinity to community structure. In addition, we discuss several emerging issues in the field (linking function to newly discovered phyla and microbial dark matter), which illustrate the changing paradigm that is seen as technology has rapidly advanced in the study of these extreme and evolutionally significant ecosystems.

## 1. Introduction

Microbial mats are a unique ecological niche representative of early life on Earth. This is due, in part, to the persistence of fossilised mat counterparts, stromatolites, which date back over 3 billion years [1]. Holistically, microbial mats are laminated organo-sedimentary biofilms which house specialized consortia of bacteria and archaea [2,3,4]. Rapid nutrient cycling across microgradients coupled with putative niche differentiation within mat layers enables diverse metabolic processes to occur in spatial proximity. These conditions thereby create an environment ideal for the establishment of unique community symbioses and cross-genera communication. In light of harsh abiotic factors, these relationships enhance consortia survival that would be less likely for individual species alone [2,5].

On a broad scale, these early ecosystems are posited to have played a major role in the oxygenation of the atmosphere, and thereby paved the way for evolution of oxygen-dependent life [6,7]. Beyond this anoxic origin, there is also the suggestion that early oceans were more saline [8], and thus early stromatolitic microbial ecosystems may have formed in hypersaline conditions. Microbial mats are therefore of great interest in the study of how microorganisms in the Precambrian period may have adapted and survived on an evolutionary scale, and for furthering our understanding of the role these primordial communities may have played in global biogeochemical cycling. In mapping the complex biotic and abiotic interactions within hypersaline microbial mats, ecological and functional models of the mat consortia can be explored, to investigate adaptations and evolution in such an extreme environment.

The understanding of microbial mat ecology has recently been deepened by the continued development of molecular methods. Pioneering studies focused on the morphology of microbial mats [9,10], or an examination of the abiotic environment including oxygen, sulfide, and light gradients, e.g., [11,12]. This initial research included a closer analysis of phylogeny targeted at the isolation of cultivatable, key players which are known to operate within multilayered consortia, namely cyanobacteria, sulphate-reducing bacteria and anoxygenic phototrophic bacteria [13,14,15,16]. As a result, functional studies typically reflected phylogenetic determinates, and were limited to oxygenic/anoxygenic photosynthesis by cyanobacteria/filamentous anoxygenic phototrophs (FAPs), aerobic/anaerobic degradation by aerobes/fermenters, sulphate reduction by sulphate-reducing bacteria, and sulphide oxidation by green/purple sulphur bacteria [17].

However, with the advent of high throughput sequencing technology, and the “omics revolution”, knowledge of microbial mats has leapt into the molecular and genomic era, revealing niche differentiation events as well as novel microbes and metabolic pathways [18,19]. It suggests our understanding of these hypersaline ecosystems is continually evolving, as the methods to study them advance. This review is designed to briefly summarize current approaches (with an emphasis on molecular techniques) and discoveries in relation to the untapped potential of hypersaline microbial mats from several well-known settings, and proposes future study directions using a combination of different contemporary techniques that can also apply to a broad range of “extreme” environments.

## 2. Microbial Diversity in Representative Hypersaline Mats

**Figure 1:**
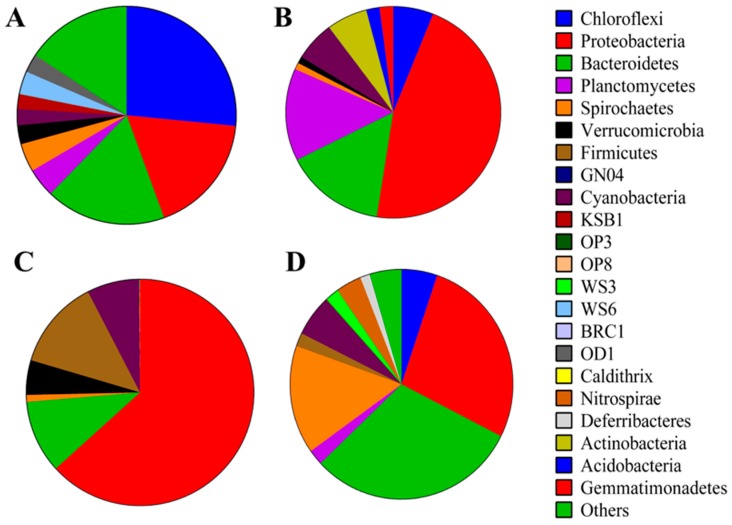
Bacterial diversity of hypersaline mats in (**A**) Guerrero Negro; (**B**) Shark Bay; (**C**) S’Avall; (**D**) Kiritimati Atoll. Proteobacteria are a major member in all four mats while Guerrero Negro is dominated by Chloroflexi, and Kiritimati Atoll dominated by Bacteroidetes. Both Shark Bay and S’Avall mats harbor abundant Proteobacteria. Data obtained from references [2,20,21,22].

The following is a short description of microbial mat communities—with a focus on the 16S rRNA gene as a marker of diversity—in select hypersaline environments. The purpose is not to describe in detail all hypersaline microbial mats—as this is beyond the scope of this review—but to provide brief selected examples and context with the emerging issues that have arisen as technology has advanced in the field. We have focused on four well-studied environments: Guerrero Negro, Shark Bay, S’Avall, and Kirrimati Atoll. Figure 1 compares the bacterial composition (based on 16S rRNA gene data) across the four hypersaline mats, in which Proteobacteria is a major member across all four samples, in particular Shark Bay and S’Avall [2,20,21,22,23]. Guerrero Negro is dominated by Chloroflexi while Bacteroidetes predominates the microbial community in Kiritimati Atoll mats. Uncultured lineages of Bacteroidetes predominated the surface layers, suggesting potentially novel phototrophic pathways in Kiritimati Atoll [20]. S’Avall is the only hypersaline mat with no reported Chloroflexi, suggesting anoxygenic phototrophy may not be as prominent in these systems, or at least conducted by other community members [21]. The difference in phototrophic pathways is suggested to cause distinct microbial interactions which leads to different assembly patterns of disparate microbial communities [24]. Kiritimati Atoll mats had lower microbial diversity compared to those in Guerrero Negro, and it was suggested that this may be related to the higher salinity in Kiritimati Atoll [20].

In terms of archaeal distribution, Guerrero Negro mats are the only systems dominated by Crenarchaeota, while the other hypersaline mats are dominated by Euryarchaeota [25]. It is important to note that the publications on the hypersaline mats reviewed here do not contain information on newly reported archaeal phyla (discussed later), and thus comparison in the current review is only based on phyla reported in these publications. Further details on individual aspects of microbial diversity (e.g., lower abundance of bacterial nitrifiers in Shark Bay) and suggestions as to some of the underlying reasons certain groups predominate in a given system are given below. The difference between the microbial structures may be a result of different physiochemical conditions such as salinity, causing distinguishable microbial assembly that likely not only affects ecosystem function, but may also determine whether lithification is going to take place.

**Guerrero Negro.** One of the most extensive and well-studied systems of hypersaline microbial mats is found in Guerrero Negro, Baja, Mexico. These mats are exposed to a range of salinities (47–125 PSU (Practical Salinity Units)), and have been characterized in detail in terms of microbial mat structure and biogeochemical cycling [7,26,27,28,29,30,31]. Halite crystallization and formation of gypsum-halite matrices were observed [32]. Early work proposed that microbial mats have a steep geochemical gradient at a millimeter scale, with oxygen decreasing rapidly with depth while sulfide increases with depth [33]. Ley and colleagues [22] conducted the first study to examine hypersaline microbial mats at successive mm intervals with depth. Total DNA was extracted and amplified by traditional 16S rRNA gene PCR, cloned, and sequenced. This study found that cyanobacteria dominated the surface layer, while Proteobacteria, Chloroflexi and Bacteroidetes were enriched in the deeper, anoxic parts of the mats [22,23]. Cyanobacteria were represented by *Microcoleus*, while other phyla (Chloroflexi, Bacteroidetes, Spirochates) have novel lineages at family to genus levels that were poorly described [23]. Of known archaeal sequences, archaea were represented by *Halorubrum* and *Methanohalophilus*, indicating potential methanogenesis in the mats [25]. Furthermore, a range of novel bacterial candidate phyla were found, designated GN01 through to GN15. Euryarchaeotes dominated the archaeal community at the surface, which decreased with depth while Crenarchaeotes dominated the anoxic parts of the mat [25]. In addition to the discovery of novel microbial diversity, Harris and colleagues reinforced that the mats are phylogenetically stratified with depth, according to light and geochemical gradients throughout the mats [22,23]. This approach is also useful to facilitate inferences of function of novel candidate phyla, e.g., novel candidates such as GN04 and Hyd24-12 are abundant in the deep, anoxic zone of the Guerrero Negro mats, suggesting anaerobic metabolisms [23].

**Shark Bay.** Hamelin Pool, located in Shark Bay, Australia, is a hypersaline pool (>60 PSU), which harbours microbial mats and stromatolites that are considered as potential modern analogues of ancient stromatolites dating back to the Archean period, e.g., [34,35]. Hamelin Pool has arguably the most extensive and diverse system of marine microbial mats [34]. Over the last 10 years, several microbial studies on these systems have been undertaken, with methods ranging from microbial isolation, 16S rRNA gene cloning, lipid analyses, to shotgun metagenome analyses, e.g., [2,3,35,36,37]. Early studies in Shark Bay utilising a clone library approach indicated these hypersaline microbial mats were dominated by Proteobacteria and Bacteroidetes [2]. Of particular interest Shark Bay appears to be novel compared with other marine microbial mat ecosystems that it contains a relatively high abundance of Archaea [3]. Euryarchaeota were the predominant members of these mats, primarily members of the haloarchaea that are known to possess high tolerance to hypersalinity [3,36]. Several novel archaea—namely *Halococcus* *hamelinensis*, *Haloferax* *elongans*, and *Haloferax* *mucosum*—were also isolated and characterised from these systems [38,39]. Several studies examining the stress response of *Halococcus* *hamelinensis* were carried out, including adaptation to high UV and salinity [40,41,42]. Interestingly, potassium uptake was not observed [40], a common adaptive response to salinity stress in archaea. Instead this archaeon demonstrated a high abundance of acidic amino acids in its proteome and a preference for glycine betaine accumulation as an osmoadaptive strategy [40,43]. Recent findings in our lab also suggests phylogenetic stratification of microbial communities at the millimeter scale in Shark Bay microbial mats, and it is likely salinity is one of the key factors shaping microbial community structure in these systems.

**S’Avall, Mallorca Island.** S’Avall solar saltern is a crystallizer pond in the Mediterranean with salt concentrations up to 35% *w*/*v* [21]. Halite is the main mineral found overlying the microbial mats. With exceptionally high sulphate concentration (> 150 mM), it is expected to harbor an abundance of sulphate reducing bacteria. 16S rRNA gene sequencing revealed that both the surface and the core of the mat are indeed dominated by Deltaproteobacteria, represented by *Desulfohalobiaceae*, an extremely halophilic sulphate-reducing bacterium [44]. Other bacterial members include Alphaproteobacteria, Cyanobacteria, Gammaproteobacteria, Bacteroidetes and Firmicutes [21]. Metagenome end-sequence analysis revealed that the mat has a high novelty of archaea with low degree of sequence similarity [45]. The archaeal community was dominated by Halobacteriales, Methanomicrobia, Thermoplasmata and Thermoprotei. However, 16S rRNA gene libraries only yielded a single phylotype in abundance, MSBL1, a novel candidate of methanogens [21]. This archeon is considered ecologically important as methanogenesis in hypersaline environments is typically outcompeted by sulphate reduction thermodynamically [46,47,48,49,50]. With higher than usual sulphate concentration and the dominance of sulphate reducing bacteria, it is still unclear how MSBL1 can effectively carry out methanogenesis at such high salinities—perhaps through the use of non-competitive substrates.

**Kiritimati Atoll.** Lake 21, Kiritimati Atoll located in the Central Pacific is an evaporitic, hypersaline lake (average salinity 170 g/L) that harbours unique mineralising mats [20,50,51]. In the lake, high concentrations of calcium and carbonate ions result in CaCO_3_ mineral supersaturation [20]. Microbial mats were precipitated with calcite, gypsum and aragonite respectively [50]. The mats were characterized with nine layers separated by different colors, and was further classified into three zones according to different types of mineral precipitation, namely the calcification zone, gypsum zone and microbialite zone. Phylogenetic studies based on large-scale analysis of 16S rRNA gene sequences have revealed that the microbial community consists of primarily Cyanobacteria, Proteobacteria, Bacteroidetes, Chloroflexi and Nitrospira, while the surface mat layers were mainly composed of halophilic phototrophs (oxygenic and anoxygenic) as well as aerobic heterotrophs [20]. Bacteroidetes, represented by *Salinibacter* and *Saprospiraceae*, dominated the surface layers, which were suggested to be photoheterotrophic [20]. Nitrospira, a nitrite-oxidizing bacterium was found in high abundance at the bottom (*ca.* 10 cm) in the anoxic zone of the mats, indicative of potentially important nitrogen cycling at this depth. The archaeal community was dominated by Halobacteria and Thaumarchaeota, and also included numerous uncultured taxonomic lineages such as Marine Benthic Group D (MBGD) and Deep Sea Hydrothermal Vent Group 6 (DHVEG-6). However, the processes of lithification, whether caused by specific metabolic traits or specific physiochemical conditions, are still unknown. Interestingly, an isolate with closest match to *Halococcus hamelinensis*—an archaeon which was previously only found in Shark Bay [38]—was recently isolated from the Kiritimati Atoll mats [4]. The three distinguishable mineralization zones were thought to be the result of the interplay between different functional guilds [4], though this needs to be confirmed as to date community metagenomics analyses have yet to be performed in Kiritmati Atoll mats.

## 3. Metagenomic Studies in Representative Hypersaline Mats

While an important first step, examining microbial community structures (16S rRNA sequences) alone is not sufficient to elucidate habitat-specific functional pathways. Metagenomics, however, is able to assign functional profiles at the community-wide level. Metagenomics utilizes taxonomic and functional binning to cluster similar nucleotide patterns together, assigning taxonomic and metabolic functions to the sample population [52]. A range of techniques has been employed (*i.e.*, G + C content, short oligomer (kmer) frequency, tetramer frequency and read coverage) to assign sequence fragments of the metagenome into microbial populations [52,53,54]. The following summarizes some of the major findings from metagenomics studies performed on the hypersaline microbial mat systems reviewed here.

**Guerrero Negro.** Shotgun metagenomic sequencing has revealed insights into the functional genetic potential of the Guerrero Negro mats. Photosynthesis-related genes were abundant in the top layers in these mats, while genes encoding ferredoxins, sulfatases and sugar degradation metabolisms (glycolysis, pentose and uronic acid degradation) were in abundance in the anoxic part of the mat [55]. This may indicate the heterotrophic metabolism of sulphated organic compounds and sugars are the main strategy for microorganisms to obtain energy in the anoxic zones in the Guerrero Negro microbial mats. This study found that millimeter-scale functional profiles were consistent with the physiochemical profile of the mat [55]. There was the suggestion by one study of acid-shifted isoelectric point amino acids detected in the Guerrero Negro mats, which were potentially caused by the enrichment of the amino acid aspartate [55]. It was proposed that this is a strategy utilized by the Guerrero Negro microbial community to adapt to their hypersaline environment, as it is known that this enrichment of amino acids allows proteins to function even in high salt concentrations [56]. However revaluation of this data suggests there may not be such a prevalence of acid shifted amino acids in these hypersaline mats as first thought [57], thus clarification is required as to whether this is indeed an adaptive strategy in the Guerrero Negro mats. Further experiments regarding putative novel functional pathways of the candidate members have also yet to be undertaken in this system.

**Shark Bay.** One recent study has delineated for the first time the functional metagenomic potential of the hypersaline mats in Shark Bay [3]. Analysis of Shark Bay mat metagenomes identified several putative osmoadaptive traits, with the majority of the sequences categorized as betaine biosynthesis and choline and betaine uptake [3]. It is likely that compatible solutes play major roles in osmoadaptive mechanisms in these communities, and this is further supported by studies on adaptation to salt in individual isolates from this environment described earlier [40,58]. It was also suggested that compatible solute turnover in the Shark Bay mats may be important both as an adaptive response as well as potential sources of energy and carbon under changing environmental conditions [3]. A range of metabolic pathways involved in sulphur cycling were identified, with the sulphur species delineated also found coinciding with cyanobacterial photosynthesis, suggesting metabolic cooperation at the surface [3,59]. Of further interest to ecosystem function, a lower abundance of common bacterial nitrifiers (*i.e.*, *Nitrosomonas*, *Nitrobacter)* was observed in the Shark Bay mats [3]. Hypersalinity has been shown in other systems to inhibit the growth of nitrifying bacteria [60], and it may be that salinity is an important factor regulating the nitrogen cycle in hypersaline settings such as the Shark Bay mats.

**S’Avall, Mallorca Island.** Metagenomic analyses were undertaken on the hypersaline microbial mats in the S’Avall solar saltern, and results indicated that carbohydrate degradation pathways (glycolysis, pyruvate metabolism and citrate cycle) were over-represented, followed by amino acid metabolism [45]. Result suggests that heterotrophic carbohydrate metabolism is the main strategy for microbial members to obtain energy, while the enrichment of amino acid metabolism is suggested to help maintain growth of the halophilic microbiota, since amino acids can also act as compatible solutes [45,56]. However, functional genes of MSBL1 (an organism described earlier as being in abundance in these systems) could not be accurately deduced due to the low degree of similarity of the annotated open reading frames [21].

Metagenomic studies from microbial mats in Shark Bay, Guerrero Negro and S’Avall have yielded similar findings of acidic amino acid enrichment, while glycine betaine uptake/biosynthesis was observed only in Shark Bay. This indicates hypersalinity is likely a selective pressure on the mat communities and amino acid enrichment may be a common osmoadaptive strategy. Heterotrophic sugar degradation pathways were found as the major metabolism in Guerrero Negro and S’Avall [21,55], while both Guerrero Negro and Shark Bay have considerable abundance of genes related to sulphur metabolism [3,55]. These putative differences in preferred metabolism and metabolic cooperation may be a reason for different microbial assemblies and thus distinct community structure.

## 4. Emerging Issues

The major pitfall of metagenomic data from microbial communities such as described above lies in the existence of newly discovered “rare phyla”, for which corresponding reference 16S rRNA gene sequences are not available in databases [61]. Thus only sequences of predominant (and therefore abundant) members in the communities will be captured and reconstructed, thus neglecting low abundance novel microbial groups [61]. Furthermore, binning of contigs into population-level is a major challenge with the absence of related reference genomes especially with novel lineages [53], referred as “microbial dark matter”, which will be discussed below.

Thus despite the advances in technology that have facilitated a greater understanding of hypersaline mats at the molecular level, the field is moving rapidly and a number of issues are important to consider when analyzing these systems.

**Correlating function with phylogeny.** A logical approach to developing models of microbial mats systems is to correlate specific metabolic pathways to the microbial taxa, thereby assigning function(s) to a specific taxon or defined Operational Taxonomic Unit (OTU). This, in turn, leads to deciphering the likely functional roles of novel phylogenetic groups and any potential evolutionary significance within the studied ecosystem. Naturally, cultivation-dependent methods remain in high use due to their capacity to generate valuable microbial mass for isolation and genetic analysis [4,18]. However, as with historical studies there remains an uncultured candidate majority that modern studies have attempted to divulge via novel culturing techniques.

A recent study on the Kiritimati Atoll microbial mats successfully isolated 62 strains that cover a broad range of microorganisms, that have been used for genomic studies to assign known metabolic functions, thus correlating function with phylogeny [4]. However the dominant microbes identified previously in this system using 16S rDNA data including Chloroflexi, Nitrospira, Thermoplasmata, Thaumarchaeota and novel candidate phyla (Hyd24-12, OP9, WS3) could not be cultivated. Thus any putative metabolic pathways of the novel candidate phyla have yet to be determined. Furthermore, despite bioinformatics tools (*i.e.*, Picrust, Tax4Fun) which can predict functional roles based on phylogenetic data, function-related information on the recently proposed super-phyla (e.g., TACK, DPANN [18,62,63] cannot be provided due to a need for (and lack of) available reference data in the corresponding databases. As a result, a functional model of microbial diversity such as in the Kiritimati Atoll mats, amongst others, has been hindered by insufficient *de novo* genomics of candidate phyla.

**Microbial dark matter (MDM).** This discussion naturally leads into addressing the aforementioned bottle-neck in environmental microbiology: “microbial dark matter” (MDM). MDM was coined as a term to denote the uncultured microbial majority that represents over half of the lineages of bacteria and archaea [18,61,64,65,66]. MDM includes microorganisms with unknown ecological roles and metabolic capability, in which cultivation efforts have not succeeded. To date, only cultivation-independent methods have detected members of MDM, e.g., [18,62,63,66,67,68], though even this in itself has some weight in the illumination of the role of MDM in microbial ecology in general, as well as specifically in hypersaline mat systems.

Of particular interest in light of this discussion is the rapidly changing shift in our understanding of the phylogenetic diversity of bacteria and archaea [18,62,63,69]. One study recently clustered newly identified bacteria with small genome size into a group described as the candidate phyla radiation (CPR) [62]. This group has successfully reconstructed complete genomes of Parcubacteria (OD1), Microgenomates (OP11), Saccharibacteria (TM7), Berkelbacteria (ACD58) and the Kazan phyla, expanding genomic information in the bacteria domain [62]. Comparative metagenomic studies support the proposition of an archaeal phylum, the TACK super-phylum (including Thaumarchaeota, Aigearchaeota, Crenarchaeota, Korachaeota and a newly proposed phylum Bathyarchaeota) [63,70,71] as well as the DPANN archaeal super-phylum (consisting of Diapherotrites, Parvarchaeota (also Micrarchaeota), Aenigmarchaeota, Nanoarchaetoa and Nanohaloarchaeota) [12,63,65,69]. Furthermore, two new archaeal phyla, Woesearchaeota and Pacearchaeota, have also recently been defined [63]. This development had refined phylogenetic estimation (predicting a target microorganism’s position on a phylogenetic tree) and expanded the archaeal and bacterial domains, leading to the need of further functional profiling of the newly proposed phyla.

Prior to these discoveries, identification of archaeal members in hypersaline microbial mats were restricted to three main phyla: Euryarchaeota, Crenarchaeota, and Thaumarchaeota respectively, e.g., [2,3,20,22,36]. As a result, the expanding genomic datasets continue to reveal further phylogenetic differentiation and novel clade formation at a phylum level. However the consistent issue of phylum level phylogenetic differentiation and metabolic/environmental interaction between co-localized populations in extreme environments remains a pertinent question to address, especially in the context of adaptation in extreme environments [72].

## 5. Combined Approaches for Molecular Ecology

Generally, it is often ideal to utilize multiple approaches to facilitate a more comprehensive view of complex microbial communities, thereby expanding our understanding of interactions and adaptations, such as those evident in hypersaline mat ecosystems. Examples of emerging approaches and their potential application in hypersaline mat systems are discussed below.

**Niche differentiation.** Niche differentiation is a strategy for microorganisms to orient themselves in the microbial community, either by interacting with other microorganisms and/or adaptation to environmental factors. The spatial arrangement and relationship between microorganisms may also reveal resource utilization patterns and metabolic specialization [73]. Furthermore, by recording the spatial distribution and niche segregation of phylogenetic diversity and comparing data with geochemical composition and functional biodiversity, microbial adaptation can be revealed as a major driver of ecosystem function [74]. For instance, an ecological model regarding the interrelationships between key functional groups was proposed for the Kiritimati Atoll mats based on niche partitioning [4]. The differentiation between different types of biomineralization at a millimeter scale (calcification, gypsum and microbialite zone) was suggested to be the result of the interplay between different functional microbial guilds and the corresponding nutrient and element cycling, including production and degradation of exopolymers, photosynthesis, sulfate reduction and fermentation. Metabolic processes occurring in unique niche spaces have been considered as a driver towards microbialite formation in hypersaline mats [4,20].

Bioinformatics tools including network correlation analysis of the co-occurrences of bacteria and archaea can also be useful for investigating specific niche differentiation in hypersaline mats. Documenting these potential interactions between taxa (co-occurrence patterns) across complex and diverse communities may help to ascertain the functional roles of environmental niches occupied by uncultured microorganisms [75,76,77], thus providing a potential method to indirectly investigate MDM.

Studies thus far regarding microbial niche differentiation have primarily been conducted on soil samples, and hypersaline samples over meter scales, where biogeochemical gradients change over large distances [59,74,78]. However, microbial mats are versatile ecosystems where complete nutrient and element cycles occur at a millimeter scale [12,22,23,79,80]. Consequently, a wide range of metabolic activities may impose steep chemical gradients and create niches with fine spatial resolution [55]. Microbial interactions, for instance, the exchange of certain nutrients and elements, can determine the presence of certain niches within the mat. To date, only a handful of published studies have examined potential niches at millimeter scales of hypersaline microbial mats [20,22,55].

Studies of niche differentiation in hypersaline mats have reinforced the existence of anoxic niches at the surface of hypersaline microbial mats, with a considerable amount of sulfate-reducing bacteria residing in the photic-oxic zone of the mats in Guerrero Negro and Kiritimati Atoll [20,22,23]. In addition, the localization of uncultured lineages of Bacteroidetes at the surface of Kiritimati Atoll and Guerrero Negro might suggest a phototrophic lifestyle of these phylotypes [20,23].

In addition to internal biochemical factors, physiochemical parameters, such as the chemical gradients of O_2_, H_2_S and NH_4_^+^, have been shown to regulate vertical stratification of a microbial community and this could influence niche formation in the hypersaline mat systems reviewed here [4,20,22,23]. Furthermore, a study of a hypersaline microbial mat in Salt Pond, San Salvador Island, Bahamas suggested that antagonistic interactions between bacteria from adjacent laminae also regulate niche formation and microbial stratification in addition to physiochemical properties [81].

Niche differentiation approaches have been shown to help delineate complex microbial communities, aiding the examination of microbial structures and proposal of ecological models in hypersaline microbial mats. These studies not only facilitated higher molecular resolution of the microbial community, but also provide a platform for further metagenomic and/or transcriptomic studies.

**Single cell genomics (SCG).** Single cell genomics (SCG) is the isolation of unculturable single cells from a microbial community, followed by whole genome amplification and subsequent sequencing of the genome [67,82]. Briefly, the isolated individual cells are subjected to whole genome amplification (WGA), by the most commonly used technique multiple displacement amplification (MDA). The resulting amplified DNA, known as single amplified genomes (SAG), can then be sequenced using any next-generation sequencing technology [61,82,83,84]. This alternative approach provides phylogenetic information as well as functional markers of the community, effectively linking function to a particular population [83,85]. SCG is a powerful tool to directly address the issue of MDM, in which novel genomes such as OP1, OP3, OP9, as well as the archaeal superphyla TACK and DPANN were all determined [18,61,66,86]. Furthermore, novel metabolic pathways can also be determined from the single cell genome, which helps to add further levels of detail to phylogenetic trees [81,82]. An example of this was the proposal of Nanohaloarchaea, a new archaeal class found in surface waters of hypersaline environments [87,88]. Iterative phylogenetic binning revealed composite genomes characterized by an ample amount of acidic amino acids, which is suggested to be the same “salt in” strategy observed in Guerrero Negro, Shark Bay and S’Avall [3,45,55,87] for microorganisms to adapt in hypersaline environments. Although to date there are no studies utilizing single cell genomics to examine hypersaline microbial mats, this approach could potentially be employed to investigate putative novel microbes.

However, despite being used as an effective tool, SCG provides only snapshots of the genomic content of uncultured single cells [65], and only targets one specific class or even genus within the novel phyla at a time. In addition to binning difficulties, it would therefore be impractical to attempt to obtain community-wide diversity via SCG. As a result, SCG and metagenomics can be considered complementary and have the potential to analyze samples in a broad *versus* narrow brush approach, the benefits of which are discussed in the following sections.

**Multiple “omics”.** Metagenomics, in contrast to single cell genomics, enables the analysis of an entire microbial community, thereby providing holistic data of metabolic and functional markers. This approach focuses on community-wide dynamics and interactions, complementary to SCG’s focus on data mining from a single cell [52,89]. In addition to previously discussed issues that binning and assembly of individual representative genomes from metagenomes is difficult, another hurdle is that a reconstructed genome from metagenomics databases is a composite genome and does not represent the single microorganism isolated from a specific environment [90]. This is a significant issue as the composite genome cannot reflect how individual microorganisms behave in a given environment or specific microenvironments, especially those that occur in hypersaline microbial mats due to fine spatial and functional resolution [91]. This is even more pronounced when annotating genes from phyla within microbial dark matter, leading to potential over-confidence in gene function prediction. Furthermore, despite the fact that metagenomics facilitates inferred predictions of gene function, DNA-based analyses cannot distinguish between expressed and non-expressed genes, hence this approach alone fails to reflect true metabolic activity within microbial mats [89,92,93].

However, when used in tandem with SCG, metagenomics is well complemented, as SCG genomic data of a single cell not only enables specific metabolic pathways to be constructed, but can also target the microbial dark matter that might be underrepresented in the sample [90]. Moreover, with SCG providing novel, rare phylogenetic and metabolic information, taxonomic binning for metagenomic databases can be improved (*i.e.*, TACK and DPANN phyla) [65]. Metagenomics can therefore make use of this novel data to enhance community-wide screening and more precisely predict potentially functional genes. Furthermore, SAGs only focus on the single cell genome and are independent of the genetic variations of the whole microbial community. As a result it can facilitate the detection of naturally occurring population-level diversity [83,94]. Coupling SCG and metagenomics can bridge this gap and associate population-level diversity with their specific functionalities, which is critical for investigating the process of niche partitioning and the construction of an accurate hypersaline microbial mat ecosystem model.

Beyond metagenomics is the realm of functional meta-analysis. Metatranscriptomics enables direct sequencing of gene transcripts from environmental samples, with quantitative measurements of community-wide microbial activities [65,94]. Metatranscriptomes can also identify which set of genes are being transcribed, and thereby illuminate complex regulatory or interactive gene systems. Complementary metagenomics and metatranscriptomics can therefore reveal how microbes respond and adapt to different physiochemical conditions at the population level [65,95]. This combined approach can simultaneously investigate taxonomic composition, functional content and gene expression patterns, revealing the overall functional and metabolic activities within the community. Although metranscriptomics have not been undertaken to date on the hypersaline mat systems that were the focus of this review, such analyses have been undertaken in other mat communities, e.g., [96,97], increasing our understanding of the complex interplay over a diel cycle of major phototrophic and heterotrophic pathways in these systems.

While metatranscriptomics elucidates genes that are actively transcribed and can provide valuable predictions of active metabolism, gene expression does not always correlate with protein content as it still indicates the potential for protein expression [98]. Thus metaproteomics can be employed to attempt to reveal the true functionality of mat communities and validate genome-based predictions. Although metaproteomic analyses have also not been undertaken on hypersaline mat communities to date, such analyses have been undertaken on hot spring microbial mat communities [98], revealing patterns of nutrient cycling over a diel cycle. Some proteins were also observed to have different transcriptional patterns when under different stress conditions (heat shock and starvation), thus metaproteomics is another powerful approach to examine ecosystem function in “extreme” environments.

Considering the tremendous diversity of uncultivated microorganisms, multiple omics approaches have the potential to identify large numbers of new, niche-specific gene and protein families among localised microbial communities in hypersaline microbial mats [92]. It can help decipher the interactions between hypersaline mat members and their environments, as well as strategies for adaptation and metabolic specialisation that may be employed [94]. A caveat is that all database-dependent analyses have their own inherent biases and limitations that need to be taken into account [64], and thus any conclusions on ecosystem function still need to be made with care and in context.

## 6. Conclusions and Future Outlook

**Figure 2:**
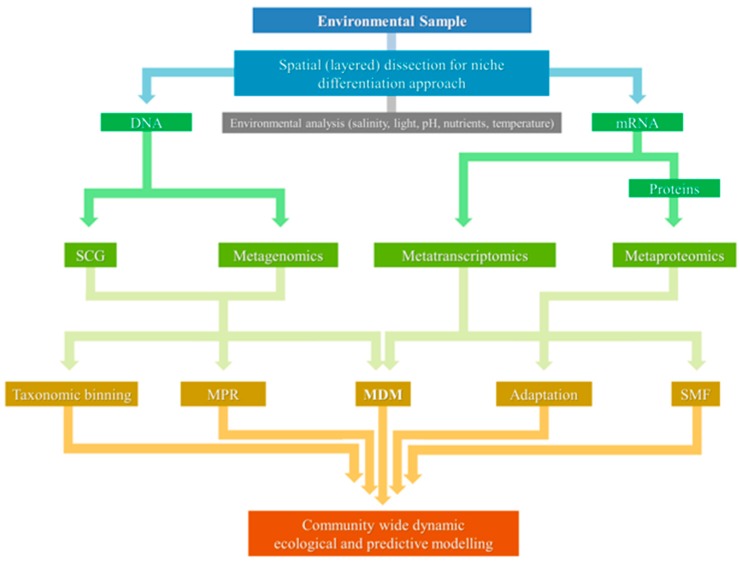
Schematic illustrating the evolving approaches and complementary pipelines in the study of hypersaline microbial mats and other “extreme” environments. MDM—Microbial dark matter; MPR—Metabolic pathway reconstruction; SCG—Single cell genomics; SMF—Specific metabolic function.

This review has sought to provide a brief overview of our current understanding of the molecular ecology of hypersaline microbial mats—with a specific focus on four disparate geographic systems—and highlighted the advances in technology that have facilitated a more holistic understanding of ecosystem function. While, for the sake of brevity, the focus in this review has been on hypersaline systems, many of the principles and approaches discussed that help delineate ecosystem function are equally applicable to microbial mats in other settings, such as those influenced by temperature and pH, e.g., [95,96,97,98,99]. In terms of future directions, major ecological roles/interactions can be further deciphered via complementary approaches [82,100], such as single cell genomics, metatranscriptomics, metaproteomics, and metabolomics (Figure 2). However it should be acknowledged that “omics” approaches also have their limitations—e.g., any sampling bias needs to be taken into account, depth of sequencing required to obtain sufficient coverage, ensuring the data is not just a “snap-shot” of the current microbial community, and considering any effects of gene transfer on community structure. Thus, to obtain the most accurate representation of overall community function in hypersaline mats, defined geochemical and physiological analyses (e.g., metabolic rates, EPS production, signaling, mineralization), as well as targeted approaches such as NanoSIMS-FISH, stable isotope probing, and qPCR of specific genes/pathways, should also be conducted to complement “omics” approaches. This will allow for a better understanding of spatial niche distribution of microbial populations within hypersaline mat environments, and can facilitate the construction of a more complete ecological model of these evolutionally significant systems.

## References

[1] Walter M.R., Buick R., Dunlop J.S.R. (1980). Stromatolites 3400–3500 Myr old from the North Pole area, Western Australia. Nature.

[2] Allen M.A., Goh F., Burns B.P., Neilan B.A. (2009). Bacterial, archaeal and eukaryotic diversity of smooth and pustular microbial mat communities in the hypersaline lagoon of Shark Bay. Geobiology.

[3] Ruvindy R., White R.A., Neilan B.A., Burns B.P. (2015). Unravelling core microbial metabolisms in the hypersaline microbial mats of Shark Bay using high-throughput metagenomics. ISME J..

[4] Spring S., Brinkmann N., Murrja M., Spröer C., Reitner J., Klenk H.P. (2015). High diversity of culturable prokaryotes in a lithifying hypersaline microbial mat. Geomicrobiol. J..

[5] Paerl H.W., Pinckney J.L., Steppe T.F. (2000). Cyanobacterial-bacterial mat consortia: Examining the functional unit of microbial survival and growth in extreme environments. Environ. Microbiol..

[6] Hoehler T.M., Bebout B.M., des Marais D.J. (2001). The role of microbial mats in the production of reduced gases on the early Earth. Nature.

[7] DesMarais D.J. (2003). Biogeochemistry of hypersaline microbial mats illustrates the dynamics of modern microbial ecosystems and the early evolution of the biosphere. Biol. Bull..

[8] Arp G., Reimer A., Reitner J. (2001). Photosynthesis-Induced Biofilm Calcification and Calcium Concentrations in Phanerozoic Oceans. Science.

[9] Logan B.W. (1961). Cryptozoon and associate stromatolites from the recent, Shark Bay, Western Australia. J. Geol..

[10] Dravis J.J. (1983). Hardened subtidal stromatolites, Bahamas. Science.

[11] Visscher P.T., Prins R.A., van Gemerden H. (1992). Rates of sulfate reduction and thiosulfate consumption in a marine microbial mat. FEMS Microbiol. Ecol..

[12] Visscher P.T., Stolz J.F. (2005). Microbial mats as bioreactors: Populations, processes and products. Palaeogeogr. Palaeocl..

[13] Bauld J., Chambers L.A., Skyring G.W. (1979). Primary productivity, sulfate reduction and sulfur isotope fractionation in algal mats and sediments of Hamelin Pool, Shark Bay, WA. Aust. J. Mar. Fresh. Res..

[14] Moriarty D.J.W. (1983). Bacterial biomass and productivity in sediments, stromatolites, and water of Hamelin Pool, Shark Bay, Western Australia. Geomicrobiol. J..

[15] Krumbein W.E. (1983). Stromatolites—The challenge of a term in space and time. Precambr. Res..

[16] Fründ C., Cohen Y. (1992). Diurnal cycles of sulfate reduction under oxic conditions in microbial mats. Appl. Environ. Microbiol..

[17] Revsbech N.P., Madsen B., Jørgensen B.B. (1986). Oxygen production and consumption in sediments determined at high spatial resolution by computer simulation of oxygen microelectrode data. Limnol. Ocenogr..

[18] Rinke C., Schwientek P., Sczyrba A., Ivanova N.N., Anderson I.J., Cheng J.F., Darling A., Malfatti S., Swan B.K., Gies E.A. (2013). Insights into the Phylogeny and Coding Potential of Microbial Dark Matter. Nature.

[19] Berg I.A., Kockelkorn D., Buckel W., Fuchs G. (2007). A 3-hydroxypropionate/4-hydroxybutyrate autotrophic carbon dioxide assimilation pathway in Archaea. Nature.

[20] Schneider D., Arp G., Reimer A., Reitner J., Daniel R. (2013). Phylogenetic analysis of a microbialite-forming microbial mat from a hypersaline lake of the Kiritimati Atoll, Central Pacific. PLoS ONE.

[21] López-López A., Yarza P., Richter M., Suárez-Suárez A., Antón J., Niemann H., Rosselló-Móra R. (2010). Extremely halophilic microbial communities in anaerobic sediments from a solar saltern. Environ. Microb. Rep..

[22] Ley R.E., Harris J.K., Wilcox J., Spear J.R., Miller S.R., Bebout B.M., Maresca J.A., Bryant D.A., Sogin M.L., Pace N.R. (2006). Unexpected diversity and complexity of the Guerrero Negro hypersaline microbial mat. Appl. Environ. Microbiol..

[23] Harris J.K., Caporaso J.G., Walker J.J., Spear J.R., Gold N.J., Robertson C.E., Hugenholtz P., Goodrich J., McDonald D., Knights D. (2013). Phylogenetic Stratigraphy in the Guerrero Negro Hypersaline Microbial Mat. ISME J..

[24] Cole J.K., Hutchison J.R., Renslow R.S., Kim Y.M., Chrisler W.B., Engelmann H.E., Dohnalkova A.C., Hu D., Metz T.O., Fredrickson J.K. (2014). Phototrophic biofilm assembly in microbial-mat-derived unicyanobacterial consortia: Model systems for the study of autotroph-heterotroph interactions. Front. Microbiol..

[25] Robertson C.E., Spear J.R., Harris J.K., Pace N.R. (2009). Diversity and stratification of Archaea in a hypersaline microbial mat. Appl. Environ. Microbiol..

[26] Minz D., Fishbain S., Green S.J., Gerard M., Cohen Y., Rittmann B.E., Stahl D.A. (1999). Unexpected population distribution in a microbial mat community: Sulfate-reducing bacteria localized to the highly oxic chemocline in contrast to a eukaryotic preference for anoxia. Appl. Environ. Microbiol..

[27] Minz D., Flax J.L., Green S.J., Gerard M., Cohen Y., Wagner M., Rittmann B.E., Stahl D.A. (1999). Diversity of sulfate-reducing bacteria in oxic and anoxic regions of a microbial mat characterized by comparative analysis of dissimilatory sulfate reductase genes. Appl. Environ. Microbiol..

[28] Risatti J.B., Capman W.C., Stahl D.A. (1994). Community structure of a microbial mat: The phylogenetic dimension. Proc. Natl. Acad. Sci. USA.

[29] Canfield D.E., des Marais D.J. (1993). Biogeochemical cycles of carbon, sulphur, and free oxygen in a microbial mat. Geochim. Cosmochim. Acta.

[30] Visscher P.T., Baumgartner L.K., Buckley D.H., Rogers D.R., Hogan M.E., Raleigh C.D., Turk K.A., des Marais D.J. (2003). Dimethyl sulphide and methanethiol formation in microbial mats: Potential pathways for biogenic signatures. Environ. Microbiol..

[31] Reimer J.J., Huerta-Diaz M.A. (2011). Phosphorus speciation and sedimentary fluxes in hypersaline sediments of the Guerrero Negro salt evaporation area, Baja California Sur, Mexico. Estuar. Coasts.

[32] Spear J.R., Ley R.E., Berger A.B., Pace N.R. (2003). Complexity in natural microbial ecosystems: The Guerrero Negro experience. Biol. Bull..

[33] Des Marais D.J. (1995). The biogeochemistry of hypersaline microbial mats. Adv. Microb. Ecol..

[34] Jahnert R.J., Collins L.B. (2012). Characteristics, Distribution and Morphogenesis of Subtidal Microbial Systems in Shark Bay, Australia. Mar. Geol..

[35] Goh F., Allen M.A., Leuko S., Kawaguchi T., Decho A.W., Burns B.P., Neilan B.A. (2009). Determining the specific microbial populations and their spatial distribution within the stromatolite ecosystem of Shark Bay. ISME J..

[36] Burns B.P., Goh F., Allen M., Neilan B.A. (2004). Microbial diversity of extant stromatolites in the hypersaline marine environment of Shark Bay, Australia. Environ. Microbiol..

[37] Allen M.A., Neilan B.A., Burns B.P., Jahnke L.L., Summons R.E. (2010). Lipid biomarkers in Hamelin Pool microbial mats and stromatolites. Org. Geochem..

[38] Goh F., Leuko S., Allen M.A., Bowman J.P., Kamekura M., Neilan B.A., Burns B.P. (2006). *Halococcus hamelinensis* sp. nov., a novel halophilic archaeon isolated from stromatolites in Shark Bay, Australia. Int. J. Syst. Evol. Microbiol..

[39] Allen M.A., Goh F., Leuko S., Echigo A., Mizuki T., Usami R., Kamekura M., Neilan B.A., Burns B.P. (2008). *Haloferax elongans* sp. nov. and *Haloferax mucosum* sp. nov., isolated from microbial mats from Hamelin Pool, Shark Bay, Australia. Int. J. Syst. Evol. Microbiol..

[40] Goh F., Jeon Y.J., Barrow K., Neilan B.A., Burns B.P. (2011). Osmoadaptive strategies of the archaeon *Halococcus hamelinensis* isolated from a hypersaline stromatolite environment. Astrobiology.

[41] Leuko S., Neilan B.A., Burns B.P., Walter M.R., Rothschild L.J. (2011). Molecular Assessment of UVC Radiation-Induced DNA Damage Repair in the Stromatolitic halophilic archaeon, *Halococcus hamelinensis*. J. Photochem. Photobiol. B.

[42] Leuko S., Goh F., Ibáñez-Peral R., Burns B.P., Walter M.R., Neilan B.A. (2008). Lysis efficiency of standard DNA extraction methods for *Halococcus* sp. in an organic rich environment. Extremophiles.

[43] Gudhka R.K., Neilan B.A., Burns B.P. (2015). Adaptaion, ecology, and evolution of the halophilic stromatolite archaeon *Halococcus hamelinensis* inferred through genome analyses. Archaea.

[44] Kjeldsen K.U., Loy A., Jakobsen T.F., Thomsen T.R., Wagner M., Ingvorsen K. (2007). Diversity of sulfate-reducing bacteria from an extreme hypersaline sediment, Great Salt Lake (Utah). FEMS Microbiol. Ecol..

[45] López-López A., Richter M., Peña A., Tamames J., Rosselló-Móra R. (2013). New insights into the archaeal diversity of a hypersaline microbial mat obtained by a metagenomics approach. Syst. Appl. Microbiol..

[46] Oremland R.S., Polcin S. (1982). Methanogenesis and sulfate reduction: Competitive and non-competitive substrates in estuarine sediments. Appl. Environ. Microbiol..

[47] King G.M., Klug M.J., Lovley D.R. (1983). Metabolism of acetate, methanol, and methylated amines in intertidal sediments of Lowes Cove, Maine. Appl. Environ. Microbiol..

[48] King G.M. (1984). Metabolism of trimethylamine, choline, and glycine betaine by sulfate-reducing and methanogenic bacteria in marine-sediments. Appl. Environ. Microbiol..

[49] Kiene R.P., Oremland R.S., Catena A., Miller L.G., Capone D.G. (1986). Metabolism of reduced methylated sulphur-compounds in anaerobic sediments and by a pure culture of an estuarine methanogen. Appl. Environ. Microbiol..

[50] Buckley D.H., Baumgartner L.K., Visscher P.T. (2008). Vertical distribution of methane metabolism in microbial mats of the Great Sippewissett Salt Marsh. Environ. Microbiol..

[51] Arp G., Helms G., Karlinska K., Schumann G., Reimer A., Reitner J., Trichet J. (2012). Formation of microbialtes on the Atoll of Kiritimati, Republic of Kiribati, Central Pacific. Geomicrobiol. J..

[52] Dröge J., McHardy A.C. (2012). Taxonomic binning of metagenome samples generated by next-generation sequencing technologies. Brief. Bioinform..

[53] Alneberg J., Bjarnason B.S., de Bruijn I., Schirmer M., Quick J., Kjaz U.Z., Lahti L., Loman N.J., Andersson A.F., Quince C. (2014). Binning metagenomics contigs by coverage and composition. Nat. Methods.

[54] Saeed I., Tang S.L., Halgamuge S.K. (2012). Unsupervised discovery of microbial population structure within metagenomes using nucleotide base composition. Nucleic Acid Res..

[55] Kunin V., Raes J., Harris J.K., Spear J.R., Walker J.J., Ivanova N., von Mering C., Bebout B.M., Pace N.R., Bork P. (2008). Millimeter-scale genetic gradients and community-level molecular convergence in a hypersaline microbial mat. Mol. Syst. Biol..

[56] Soppa J. (2006). From genomes to function: Haloarchaea as model organisms. Microbiology.

[57] Bardavid R.E., Oren A. (2012). Acid-shifted isoelectric point profiles of the proteins in a hypersaline microbial mat: An adaptation to life at high salt concentrations?. Extremophiles.

[58] Goh F., Barrow K.D., Neilan B.A., Burns B.P. (2010). Identification and regulation of novel compatible solutes from hypersaline stromatolite-associated cyanobacteria. Arch. Microbiol..

[59] Pages A., Welsch D.T., Teasdale P.R., Grice K., Vacher M., Bennett W.W., Visscher P.T. (2014). Diel fluctuations in solute distributions and biogeochemical cycling in a hypersaline microbial mat from Shark Bay, WA. Mar. Chem..

[60] Jeffries T.C., Seymour J.R., Newton K., Smith R.J., Seuront L., Mitchell J.G. (2012). Increases in the abundance of microbial genes encoding halotolerance and photosynthesis along a sediment salinity gradient. Biogeosciences.

[61] Hedlund B.P., Dodsworth J.A., Murugapiran S.K., Rinke C., Woyke T. (2014). Impact of single-cell genomics and metagenomics on the emerging view of extremophile “microbial dark matter”. Extremophiles.

[62] Brown C.T., Hug L.A., Thomas B.C., Sharon I., Castelle C.J., Singh A., Wilkins M.J., Wrighton K.C., Williams K.H., Banfield J.F. (2015). Unusual biology across a group comprising more than 15% of domain Bacteria. Nature.

[63] Castelle C.J., Wrighton K.C., Thomas B.C., Hug L.A., Brown C.T., Wilkins M.J., Frischkorn K.R., Tringe S.G., Singh A., Markillie L.M. (2015). Genomic expansion of domain Archaea highlights roles for organsims from new phyla in anaerobic carbon cycling. Curr. Biol..

[64] McDonald D., Price M.N., Goodrich J., Nawrocki E.P., DeSantis T.Z., Probst A., Andersen G.L., Knight R., Hugenholtz P. (2012). An improved Greengenes taxonomy with explicit ranks for ecological and evolutionary analyses of bacteria and archaea. ISME J..

[65] Baker B.J., Dick G.J. (2013). Omic Approaches in Microbial Ecology: Charting the Unknown. Microbe.

[66] Dodsworth J.A., Blainey P.C., Murugapiran S.K., Swingley W.D., Ross C.A., Tringe S.G., Chain P.S.G., Scholz M.B., Lo C.C., Raymond J. (2013). Single-cell and metagenomics analyses indicate a fermentative, saccharolytic lifestyle for members of the OP9 lineage. Nat. Commun..

[67] Blainey P.C. (2013). The future is now: Single-cell genomics of bacteria and archaea. Microbiol. Rev..

[68] Scholz M.B., Lo C.C., Chain P.S.G. (2012). Next generation sequencing and bioinformatics bottlenecks: The current state of metagenomics data analysis. Curr. Opin. Biotechnol..

[69] Youssef N.H., Rinke C., Stepanauskas R., Farag I., Woyke T., Elshahed M.S. (2015). Insights into the metabolism, lifestyle and putative evolutionary history of the novel archaeal phylum “Diapherotrites”. ISME J..

[70] Meng J., Xu J., Qin D., He Y., Xiao X., Wang F. (2014). Genetic and functional properties of uncultivated MCG archaea assessed by metagenome and gene expression analyses. ISME J..

[71] Guy L., Ettema T.J. (2011). The archaeal “TACK” superphylum and the origin of eukaryotes. Trends Micriobiol..

[72] Nichols D., Cahoon N., Trakhtenberg E.M., Pham L., Mehta A., Belanger A., Kanigan T., Lewis K., Epstein S.S. (2010). Use of ichip for high-throughput *in situ* cultivation of “uncultivable” microbial species. Appl. Environ. Microbiol..

[73] Grünke S., Felden J., Lichtschlag A., Girnth A.C., de Beer D., Wenzhöfer F., Boetius A. (2011). Niche differentiation among mat-forming, sulfide-oxidizing bacteria at cold seeps of the Nile Deep Sea Fan (Eastern Mediterranean Sea). Geobiology.

[74] Vigneron A., Cruaud P., Roussel E.G., Pignet P., Caprais J.C., Callac N., Ciobanu M.C., Godfroy A., Cragg B.A., Parkes J.R. (2014). Phylogenetic and functional diversity of microbial communities associated with subsurface sediments of the Sonora Margin, Guaymas Basin. PLoS ONE.

[75] Ruan Q., Dutta D., Schwalbach M.S., Steele J.A., Fuhrman J.A., Sun F. (2006). Local similarity analysis reveals unique associations among marine bacterioplankton species and environmental factors. Bioinformatics.

[76] Fuhrman J.A., Steele J.A. (2008). Community structure of marine bacterioplankton: Patterns, networks, and relationships to function. Aquat. Microb. Ecol..

[77] Chaffron S., Rehrauer H., Pernthaler J., von Mering C. (2010). A global network of coexisting microbes from environmental and whole—Genome sequence data. Genome Res..

[78] Andrei A.Ş., Robeson M.S., Baricz A., Coman C., Muntean V., Ionescu A., Etiope G., Alexe M., Sicora C.I., Podar M. (2015). Contrasting taxonomic stratification of microbial communities in two hypersaline meromictic lakes. ISME J..

[79] Ludemann H., Arth I., Liesack W. (2000). Spatial changes in the bacterial community structure along a vertical oxygen gradient in flooded paddy soil cores. Appl. Environ. Microbiol..

[80] Stomp M., Huisman J., Stal L., Matthijs H.C.P. (2007). Colorful niches of phototrophic microorganisms shaped by vibrations of the water molecule. ISME J..

[81] Long R.A., Eveillard D., Franco S.L.M., Reeves E., Pinckney L.L. (2013). Antagonistic interactions between heterotrophic bacteria as a potential regulator of community structure of hypersaline microbial mats. FEMS Microbiol. Ecol..

[82] De Jager V., Siezen R.J. (2011). Single-cell genomics: Unravelling the genomes of unculturable microorganisms. Microb. Biotechnol..

[83] Stepanauskas R. (2012). Single cell genomics: An individual look at microbes. Curr. Opin. Microbiol..

[84] Dean F.B., Hosono S., Fang L.H., Wu X.H., Faruqi A.F., Bray-Ward P., Sun Z.Y., Zong Q.L., Du Y.F., Du J. (2002). Comprehensive human genome amplification using multiple displacement amplification. Proc. Natl. Acad. Sci. USA.

[85] Walker A. (2014). Adding genomic “foliage” to the tree of life. Nat. Rev. Microbiol..

[86] Kolinko S., Jogler C., Katzmann E., Wanner G., Peplies J., Schüler D. (2012). Single-cell analysis reveals a novel uncultivated magnetotactic bacterium within the candidate division OP3. Environ. Microbiol..

[87] Narasingarao P., Podell S., Ugalde J.A., Brochier-Armanet C., Emerson J.B., Brocks J.J., Heidelberg K.B., Banfield J.F., Allen E.E. (2012). *De novo* metagenomics assembly reveals abundant novel major lineage of Archaea in hypersaline microbial communities. ISME J..

[88] Ghai R., Pašic L., Fernández A.B., Martin-Cuadrado A.B., Mizuno C.M., McMahon K.D., Papke R.T., Stepanauskas R., Rodriguez-Brito B., Rohwer F. (2011). New abundant microbial groups in aquatic hypersaline environments. Sci. Rep..

[89] Simon C., Daniel R. (2011). Metagenomic analyses: Past and future trends. Appl. Environ. Microbiol..

[90] Podar M., Abulencia C.B., Walcher M., Hutchison D., Zengler K., Garcia J.A., Holland T., Cotton D., Hauser L., Keller M. (2007). Targeted access to the genomes of low-abundance organisms in complex microbial communities. Appl. Environ. Microb..

[91] Dupraz C., Reid R.P., Braissant O., Decho A.W., Norman R.S., Visscher P.T. (2008). Processes of carbonate precipitation in modern microbial mats. Earth Sci. Rev..

[92] Sorek R., Cossart P. (2010). Prokaryotic transcriptomics: A new view on regulation, physiology and pathogenicity. Nat. Rev. Genet..

[93] Bhaya B., Grossman A.R., Steunou A.S., Khuri N., Cohan F.M., Hamamura N., Melendrez M.C., Bateson M.M., Ward D.M., Heidelberg J.F. (2007). Population level functional diversity in a microbial community revealed by comparative genomic and metagenomics analyses. ISME J..

[94] Shi Y., Tyson G.W., Eppley J.M., DeLong E.F. (2011). Integrated metatranscriptomic and metagenomics analyses of stratified microbial assemblages in the open ocean. ISME J..

[95] Chen L.X., Hu M., Huang L.N., Hua Z.S., Kuang J.L., Li S.J., Shu W.S. (2015). Comparative metagenomics and metatranscriptomic analyses of microbial communities in acid mine drainage. ISME J..

[96] Liu Z., Klatt C.G., Wood J.M., Rusch D.B., Ludwig M., Wittekindt N., Tomsho L.P., Schuster S.C., Ward D.M., Bryant D.A. (2011). Metatranscriptomic analyses of chlorophototrophs of a hot-spring microbial mat. ISME J..

[97] Mobberley J.M., Khodadad C.L.M., Visccher P.T., Reid R.P., Hagan P., Foster J.S. (2015). Inner workings of thrombolites: Spatial gradients of metabolic activity as revealed by metatranscriptome profiling. Sci. Rep..

[98] Schaffert C.S., Ward D.M., Klatt C.G., Pauley M., Steinke L.A. (2012). Identification and distribution of high-abundance proteins in the Octopus Spring microbial mat community. Appl. Environ. Microbiol..

[99] Jungblut A.D.J., Allen M.A., Burns B.P., Neilan B.A. (2009). Lipid biomarker analysis of cyanobacterial dominated microbial mats in melt water ponds on the McMurdo Ice Shelf, Antarctica. Org. Geochem..

[100] Rubakhin S.S., Romanova E.V., Nemes P., Sweedler J.V. (2011). Profiling metabolites and peptides in single cells. Nat. Methods.

